# Adolescent leisure-time physical activity and eating disorders: a longitudinal population-based twin study

**DOI:** 10.1007/s40519-024-01670-8

**Published:** 2024-06-08

**Authors:** Nadja Anis, Anna Keski-Rahkonen, Sara Kaartinen, Yasmina Silén, Jaakko Kaprio, Sari Aaltonen

**Affiliations:** 1 Department of Public Health, University of Helsinki, P.O. Box 20, 00014 Helsinki, Finland; 2 Department of Physical Medicine and Rehabilitation, HUS Hyvinkää Hospital, P.O. Box 585, 05850 Hyvinkää, Finland; 3 Institute for Molecular Medicine Finland (FIMM), University of Helsinki, P.O. Box 20, 00014 Helsinki, Finland

**Keywords:** Cohort study, Exercise, Long term, Young adults, Anorexia nervosa

## Abstract

**Purpose:**

High levels of physical activity have been documented in eating disorder patients. Our aim was to examine whether adolescent leisure-time physical activity is prospectively associated with eating disorders in adolescence and young adulthood.

**Methods:**

Finnish twins born in 1983–1987 reported their physical activity frequency at ages 12, 14, and 17. A subsample of participants underwent structured, retrospective interviews for eating disorders at the mean age of 22.4 years. Associations between female twins’ physical activity and future eating disorders (571–683 twins/wave) were investigated with the Cox proportional hazards model. To illustrate the physical activity similarity of the co-twins in a twin pair, we used cross-tabulation of eating disorder–discordant twin pairs (13–24 pairs/wave).

**Results:**

After adjusting for several covariates, we found no statistically significant longitudinal association between physical activity and eating disorders. This applied when all eating disorders were combined but also when assessed separately as restrictive and non-restrictive eating disorders. Co-twins’ physical activity in adolescence tended to be similar irrespective of their future eating disorder, supporting the results of the regression analysis.

**Conclusion:**

We observed no evidence of adolescent physical activity frequency being prospectively associated with eating disorders in female twins. Further longitudinal studies with larger sample sizes and more detailed physical activity data are needed.

*Level of evidence*: III, evidence obtained from cohort or case–control analytic studies.

**Supplementary Information:**

The online version contains supplementary material available at 10.1007/s40519-024-01670-8.

## Introduction

Eating disorders (EDs) are a common public health concern [1]: their prevalence has increased worldwide in recent years [2]. Although many factors have been recognized to be associated with risk of EDs [3], the relationship between physical activity and the onset of EDs remains poorly understood. A lack of longitudinal studies makes it difficult to infer any temporal relationship between physical activity and EDs.

A recent large systematic review summarized three decades of scientific inquiry on the relationship between morbid exercise and EDs, and only 1 of the 67 studies featured a longitudinal design [4]. This longitudinal study suggested that a higher commitment to physical activity may be part of the psychopathology of anorexia nervosa [5]. However, another longitudinal study not included in the review found that adolescents’ physical activity levels increased one year before the diagnosis of anorexia nervosa, indicating that physical activity could be part of the pathogenesis of the ED [6]. Two recent studies have also indicated that a psychological drive to exercise excessively (including exercising when sick or injured) or exercise that is specifically aimed at weight loss or gain may be associated with future ED symptoms [7, 8].

The larger body of cross-sectional studies have also suggested an ambiguous relationship between physical activity and EDs [4, 9, 10]. However, many studies have examined athletes and high-volume exercisers [11]. A lack of studies of the general population may lead to a distorted view of the role of physical activity on EDs because athletes’ physical activity routines usually differ considerably from other population groups. Moreover, only a few studies have explored associations between the frequency of physical activity and disturbed eating [12], with previous studies having explored compulsive physical activity [13] or used other generic terminology to describe exercise-related behavior [4, 14].

However, the panel of experts have recently recommended that in addition to qualitative aspects of physical activity, also the frequency of physical activity should be assessed when studying physical activity behavior in individuals with ED [15]. The reason for this is that physical activity frequency may give more information on how compulsive exercise manifests in different types of EDs. Some studies argue that among persons with non-restrictive EDs (e.g., bulimia nervosa or binge eating disorder), a belief in the need to be physically active does not necessarily imply that the actual amount of physical activity is excessive. Still, persons with non-restrictive EDs may display high scores on compulsive exercise instruments despite their objectively assessed low volume of physical activity [16]. However, in restrictive EDs (e.g., anorexia nervosa), compulsive exercise may more likely include increased levels of physical activity [15].

Defining and measuring physical activity and exercise in EDs is difficult due to patients with EDs engaging in a variety of physical activity they may not see as exercise [15]. Based on the general agreement of researchers and professionals, physical activity that is not required as an essential physical activity of daily living, is not done during working hours and is performed at the person’s discretion is called leisure-time physical activity. Exercise is a subcategory of leisure-time physical activity that is planned, structured, repetitive, and purposeful in the sense that the improvement or maintenance of one or more components of physical fitness is the objective [17, 18]. It has been suggested that it would be beneficial to assess for all types and levels of physical activity in EDs (e.g., walking for errands or standing), not just those typically considered exercise (e.g., running to improve fitness or weightlifting) [15].

The lack of longitudinal studies examining the relationship between quantitative aspects of physical activity and the onset of EDs later in life is a limitation that highlights the need for further research since the summarized evidence is entirely derived from cross-sectional data [4]. Therefore, using population-based Finnish twin data, we aimed to longitudinally investigate whether the frequency of female twins’ leisure-time physical activity at ages 12, 14, and 17 is associated with future development of EDs in adolescence and young adulthood.

Because the previous literature shows increased physical activity levels at least one year prior to anorexia nervosa, we hypothesize that leisure-time physical activity frequency (hereafter, physical activity) will demonstrate an association with future EDs.

## Materials and methods

**Fig. 1:**
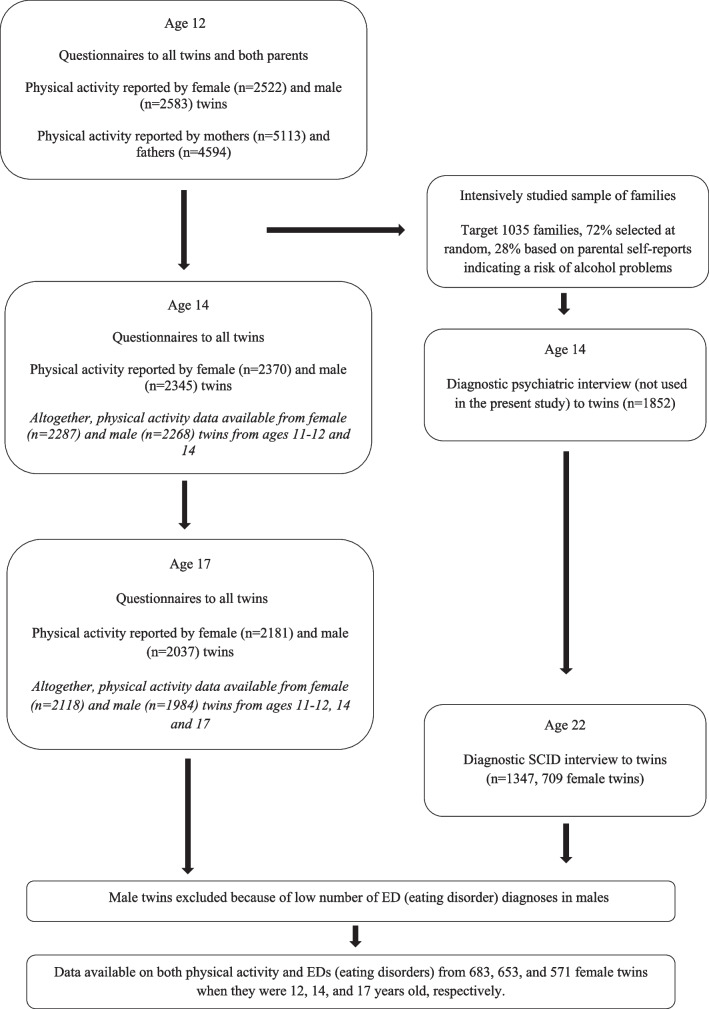
Flowchart of data collection procedures for twin families in the FinnTwin12 study. Participants in the intensively studied sample took part in interviews, as indicated on the right-hand column of the flowchart

The FinnTwin12 (FT12) study is a longitudinal study of Finnish twins born 1983–1987 and their families [19]. The twins were identified from the Central Population Registry of Finland, and the data were collected through mailed questionnaires in four waves. The response rates have ranged from 85 to 90% per data collection wave [19]. Our original data had information on female and male twins. However, we focus only on female twins as there were too few ED cases in male twins (*n* = 15, 2.4% of all). Figure 1 shows the study participation flowchart.

Altogether, 5600 twins and their families were enrolled in the FT12 study. We use physical activity reported by the female twins in the three first waves. In the first wave, the twins completed questionnaires when the twins were 11–12 years old (*n* = 2522). Twins` parents responded questions on their own physical activity at that same time point using separate maternal and paternal questionnaires. The parents did not provide any other data on their own or their children’s physical activity. The twins were surveyed again at the mean ages of 14.1 (range 14–15 years, *n* = 2370) and 17.6 (range17–19 years, *n* = 2181) [19].

In addition to the questionnaire-based data collection, there was an intensively studied sample of 1035 families participating at baseline. These families were selected at random (72.3%) or based on parental self-reports indicating a risk of alcohol problems (27.7%) [20]. At age 14, 1852 twins (89% of twin individuals in the intensively studied families) were interviewed and asked to participate in the clinical study as young adults in their early twenties (total *n* = 1347). Finally, a subsample of female twins (*n* = 709) attended a clinical interview, including assessment of EDs at the mean age of 22.4 years (range 19–26 years). We have data available on both physical activity and EDs from 683, 653, and 571 female twins when they were 12, 14, and 17 years old, respectively, with data from 561 female twins at all 3 time points.

### Assessment of leisure-time physical activity

Twins’ self-reported physical activity was based on a structured question on the frequency of physical activity (excluding physical education in school) with the response options slightly varying at different waves. The item was asked in the same form in all waves (no time specification): “How often do you engage in physical activity or exercise during your leisure time?”. At age 12, there were 5 response options: (1) not at all, (2) two to three times in six months, (3) two to three times a month, (4) two to three times a week, (5) just about every day. At ages 14 and 17, there were 7 response options: (1) not at all, (2) less than once a month, (3) one to two times a month, (4) about once a week, (5) two to three times a week, (6) four to five times a week, (7) just about every day.

To harmonize response options across the waves, and to keep our analyses more informative considering the low number of twins with EDs in the original physical activity frequency groups, we divided the twins into three categories based on the above-reported physical activity frequencies. We combined the answers of the 12-year-olds as follows: (1) “not at all–two to three times a month”, (2) “two to three times a week”, (3) “just about every day”. At the age 14 and 17, the division was: (1) “not at all–about once a week”, (2) “two to three times a week”, (3) “four to five times a week–just about every day”. We named these classes as (1) Rarely, (2) Regularly, and (3) Daily while recognizing the differences between assessment ages. The original and revised physical activity categories are shown in Supplementary Table 1 and Table 1, respectively.

**Table 1:** Wald test results on the physical activity of the study participants by their future eating disorder (ED) status

Characteristics	Future ED status ED dx *n (%)*	No ED dx *n (%)*	*p*-value
Physical activity at age 12			
Rarely	*51 (42.1%)*	270 (48.0%)	0.23
Regularly	*58 (47.9%)*	248 (44.1%)	
Daily	*12 (9.9%)*	44 (7.8%)	
Physical activity at age 14			
Rarely	*24 (26.1%)*	208 (37.1%)	**0.03**
Regularly	*31 (33.7%)*	176 (31.4%)	
Daily	*37 (40.2%)*	177 (31.6%)	
Physical activity at age 17			
Rarely	*15 (32.6%)*	199 (37.9%)	0.68
Regularly	*18 (39.1%)*	175 (33.3%)	
Daily	*13 (28.3%)*	151 (28.8%)	

Statistically significant results are in bold

ED: Eating disorder; dx: diagnosis

### Assessment of eating disorders

When the twins were at the mean age of 22.4 years (range 19–26 years), interviews for EDs were conducted using Structured Clinical Interview for Diagnostic and Statistical Manual of Mental Disorders, fourth edition (SCID) [21]. During the SCID interview, twins were questioned in detail about their eating behaviors, compensatory habits, and potential cognitive distortions related to food, weight, and body image by healthcare professionals. Twins were asked to elaborate on the time course of symptoms, detection in healthcare, and any treatment related to EDs. Additionally, twins’ height and weight were measured or self-reported (61.5% and 38.5%, respectively), and they were asked about their weight history. Based on the collected information, three medical doctors highly experienced in the diagnosis and treatment of EDs established consensus Fifth Diagnostic and Statistical Manual of Mental Disorders (DSM-5) ED diagnoses [1]. Most of the participants were diagnosed with an ED for the first time through this assessment. Only 32% (*n* = 41) of females diagnosed with ED in the SCID interviews had their condition detected in healthcare [22]. There were no participants with a previous or current ED diagnosis that would not have been detected with the SCID interview. A dichotomized ED variable (yes ED/no ED) and the age of ED onset were used in the analyses.

In total, 127 female twins (17.9% of all female twins) were diagnosed with ED as defined by the DSM-5. Twins’ age at the time they were diagnosed with ED ranged from 9 to 22 years, and the onset peak of EDs was at age 16–19. Two female participants had two ED diagnoses. The diagnoses were not made simultaneously but at different time points, and in both cases the diagnoses were anorexia nervosa and bulimia nervosa. The prevalence of DSM-5 EDs were 6.2% (*n* = 44) for anorexia nervosa, 2.4% (*n* = 17) for bulimia nervosa, 0.6% (*n* = 4) for binge eating disorder, 4.5% (*n* = 32) for other specified feeding/eating disorders, and 4.5% (*n* = 32) for unspecified feeding/eating disorders [1].

### Statistical methods

We performed analyses using the Stata statistical software, version 15.1 [23]. First, we produced descriptive statistics for physical activity variables at each wave. By using the Wald test, we compared female adolescent physical activity frequencies by future ED status, as well as examined whether there was a difference in physical activity changes (decrease, stability, or increase) between ages 12 and 14 in relation to ED status after age 14 or in physical activity change between ages 14 and 17 in relation to ED status after age 17.

The association between twins’ physical activity and future EDs was investigated in all study waves with the Cox proportional hazards model. The follow-up started at age 12, 14, or 17 depending on the study wave and ended at the diagnosis of ED or time of last examination. Two participants were removed from the regression analysis because their ED onset ages were not recorded. Age was the time variable in the model, hence we did not need to adjust for age. Because of the low incidence of any specific ED diagnosis in our data, we combined and analyzed all EDs together as main results and restrictive (e.g., anorexia nervosa, atypical anorexia nervosa) and non-restrictive (e.g., bulimia nervosa, binge eating disorder) as additional results. With these additional analyses, we examined whether physical activity is differently associated with future ED development between the groups, as previous studies have suggested that divergence exists in the underlying etiology between restrictive-type EDs and bulimic spectrum disorders [3]. Twins in the least active group were used as the reference group, and hazard ratios were presented with 95% confidence intervals.

In adjusted models, based on the previous literature on EDs [24, 25] and physical activity behavior [26], as well as based on our testing on confounding effects, we considered the following factors that could impact physical activity and EDs: adolescent academic performance, parental educational level, and both maternal and paternal physical activity. Twins’ academic performance was assessed as a teacher-reported grade point average at ages 12 and 14 and as a self-reported student status at age 17. Parental education and physical activity levels were measured by questionnaires when the twins were 12 years old. We calculated leisure-time metabolic equivalent of task hours per day (ltMETh/d) values to measure the total energy expenditure of the parents’ physical activity (details in Supplementary Material 1 and Supplementary Table 2).

Before the analyses, the Cox proportional hazards assumptions were tested by using Schoenfeld residuals (ph-test in Stata) and by plotting. Because we found parental education to violate the proportional hazards assumptions in non-restrictive ED analyses at age 12 and in all, restrictive and non-restrictive ED analyses at age 14, we stratified these Cox models by this covariate. After these stratifications, the models fulfilled the assumptions.

We carried out the analyses considering twins as individuals. When twins are analyzed as individuals, the observations, and their error terms between co-twins of a twin pair may be correlated. Therefore, Stata’s cluster option was applied to account for the lack of statistical independence of the two observations within a pair [27].

To support our regression analyses, we illustrated the potential association between physical activity and ED variables among female twin pairs by cross-tabulations. The rates of concordance and discordance in twin pairs were utilized: if twin pairs discordant for EDs are also discordant for adolescent physical activity (double-discordance), it may imply a potential association between physical activity and EDs. Only those female twin pairs who had an extreme discordance between their physical activity frequencies (i.e., rarely versus daily) were considered as physical activity discordant and were cross-tabulated.

## Results

No differences in adolescent physical activity were observed between those female twins with or without future EDs, except at age 14 (Table 1). Twins with future EDs were more likely to be physically active at age 14 than those without future EDs (*p* = 0.03). Overall, twins with future EDs reported their physical activity frequency slightly more often than their healthy co-twins at age 12 (*p* = 0.04), but no differences were found at ages 14 and 17 (*p* = 0.08 and *p* = 0.82, respectively). When we further compared those female twins who had ED after age 14 or age 17 to those who did not, we found no statistically significant difference in change in twins’ physical activity behavior from age 12 to age 14 (*p* = 0.63), or from age 14 to 17 (*p* = 0.22), respectively.

In the unadjusted Cox proportional hazards model, we found a statistically significant longitudinal association between adolescent daily physical activity and future development of EDs in female twins at age 14 (*p* = 0.04), but this association vanished after adjusting for participants’ academic performance, parental education, and physical activity (Table 2). In the adjusted model, no covariates, except academic performance at age 14 (*p* < 0.001) were significantly related to future development of EDs.

**Table 2:** Longitudinal association of female adolescents’ physical activity and eating disorders (EDs) based on Cox proportional hazards model (including unadjusted and adjusted model)

	Unadjusted model^a^					Adjusted model^b^				
	Future ED					Future ED				
	ED dx	No ED dx	HR	95% CI	*p*-value	ED dx	No ED dx	HR	95% CI	*p*-value
Physical activity at age 12	*N* = 121	*N* = 562				*N* = 105	*N* = 466			
Rarely	51	270	1			49	232	1		
Regularly	58	248	1.19	0.79–1.77	0.41	46	201	1.17	0.59–2.32	0.65
Daily	12	44	1.35	0.74–2.47	0.33	10	33	2.01	0.72–5.64	0.18
Physical activity at age 14	*N* = 92	*N* = 561				*N* = 64	*N* = 377			
Rarely	24	208	1			19	138	1		
Regularly	31	176	1.51	0.88–2.59	0.13	20	120	1.15	0.60–2.21	0.68
Daily	37	177	1.71	1.02–2.88	**0.04**	25	119	1.34	0.73–2.48	0.34
Physical activity at age 17	*N* = 46	*N* = 525				*N* = 41	*N* = 463			
Rarely	15	199	1			14	172	1		
Regularly	18	175	1.35	0.69–2.63	0.39	15	154	1.11	0.55–2.23	0.78
Daily	13	151	1.14	0.55–2.36	0.72	12	137	0.95	0.47–1.94	0.89

Statistically significant results are in bold. Covariates: school performance (grade point average at age 12, grade point average at age 14) or student status (student status at age 17), maternal physical activity (ltMETh/d), paternal physical activity (ltMETh/d), parental education level based on the level of the more highly educated parent

ED: eating disorder; dx: diagnosis; CI: confidence interval; HR: hazard ratio

^a^Model without covariates

^b^Model adjusted for covariates (all ages) and at age 14 also stratified by parental education level

No other associations were detected. No significant longitudinal associations with physical activity were found when EDs were analyzed as restrictive and non-restrictive ED groups (Supplementary Tables 3 and 4).

The similarity of the co-twins of a twin pair in terms of physical activity and EDs are illustrated by cross-tabulations in Table 3. The female twin pairs discordant for EDs (one co-twin of a twin pair has a future ED diagnosis and the other does not) were mostly concordant regarding their adolescent physical activity in each study wave, further supporting our regression results that physical activity and EDs are not associated.

**Table 3:** Cross-tabulations of physical activity of the eating disorder (ED)-discordant female twin pairs

	Co-twin without future ED Physical activity at age 12	
	Rarely	Daily
Co-twin with future ED Physical activity at age 12		
Rarely	17	1
Daily	2	4

	Physical activity at age 14	
	Rarely	Daily
Co-twin with future ED Physical activity at age 14		
Rarely	8	0
Daily	3	13

	Physical activity at age 17	
	Rarely	Daily
Co-twin with future ED Physical activity at age 17		
Rarely	6	0
Daily	1	6

ED: eating disorder

The numbers in the table represent the number of twin pairs

## Discussion

The current study aimed to examine whether the frequencies of physical activity at ages 12, 14, and 17 are associated with future development of EDs in adolescence and young adulthood in a population-based longitudinal study of twins. We found no associations between adolescent physical activity and future development of EDs in female twins, after adjusting for participants’ academic performance, parental education, and physical activity. This was contrary to our hypothesis. We were also able to illustrate that female twin pairs discordant for EDs were not discordant for their frequency of adolescent physical activity, being consistent with our regression results on individuals.

One previous study found that the level of physical activity increased one year before the diagnosis of an ED [6]. However, we found no change in physical activity from age 12 to age 14 and from age 14 to age 17 when we compared female twins with and without ED after ages 14 and 17, respectively. Our results may differ from the previous ones because our data included participants whose EDs were, in most cases, not detected by the healthcare system [22]. However, our rate of detection of EDs was like previous studies in the DSM-5 era [28–32]. Moreover, we included all EDs in our analyses, while Davis and colleagues [6] focused only on anorexia nervosa. Consequently, our results cannot be directly compared to studies that have only one specific ED in their sample. Davis and colleagues (2005) also conducted analyses using physical activity data one year prior to the onset of ED [6], while we were unable to use the exact same design because it would have resulted in a very low number of participants.

Overall, very few previous studies have been conducted considering physical activity and EDs other than anorexia nervosa [14]. Although the etiological features in EDs have been shown to be overlapping [3], physical activity routines might differ considerably between ED diagnostic groups. A recent large meta-analysis concluded that further longitudinal research should clarify the relationship between exercise and symptoms underlying specific ED [4]. Even though we could not distinguish between all specific ED diagnoses, our results of restrictive and non-restrictive EDs (although not fully reliable due to small sample size in some of the physical activity categories) hints to these ED-specific results.

In our study, twins with future EDs reported their early adolescence physical activity slightly more often than healthy twins. This suggests that individuals with future EDs were interested in reporting their physical activity, yet they may have over- or underreported their true levels of physical activity. In general, ED patients tend to underreport their physical activity levels [33]. This can make the self-reports of physical activity even less reliable among individuals with EDs. Moreover, self-reporting may have varied depending on the time of year and adolescent school attendance as the specific timing of sending the questionnaires to the twins was not consistent between the study waves. It is also possible that participants may have interpreted the time frame of our physical activity question differently, because the time frame was not specified in the question. Therefore, future research could benefit from device-based physical activity measurements. However, it is important to note that physical activity trackers are expensive and their accuracy can be questionable [34], thus limiting the use of physical activity trackers in large population-based studies.

Moreover, previous studies have suggested that exercise for both weight loss and gain [7, 8] as well as psychological drive to exercise [7], and exercise compulsiveness [35] are more likely to predict future ED symptoms. It is obvious that the definition and assessment of physical activity used in the study affects the results: the prevalence of problematic exercise has been shown to vary from 5 to 54%, depending on the number of criteria used for the definition of problematic exercise [36]. The purported role of physical activity in EDs is mostly derived from studies of athletes, which have reported varying results [11, 37]. Our findings are not comparable to athlete population studies because athletes’ physical activity routines are very different from the self-reported frequencies of physical activity in a general population. Further longitudinal research could benefit from detailed information on the compulsivity and compensatory aspects of physical activity as well as physical activity assessing the frequency, duration and intensity measured by both comprehensive questionnaires and devices to form a thorough assessment of physical activity.

## Strengths and limitations

Our study was based on self-reports of physical activity. We were not able to have detailed information on the intensity, compulsivity, and types of sport or physical activities. Nevertheless, our assessment of physical activity is valid and reliable [38, 39] and well-suited for identifying the participants’ general engagement in physical activity. Our study was also limited by the relatively small sample of some of the analyses.

However, we believe that the limitations of this study were offset by its strengths: a population-based study with a longitudinal design and the opportunity to examine twin siblings. Recall bias was minimal in our study, as the frequency of physical activity was not reported retrospectively and assessments were repeated through adolescence. In contrast to many previous studies [7, 12, 40], EDs were established with a semi-structured clinical interview by trained staff. The use of a subsample of families at high risk for alcohol problems in the clinical ED interviews could also have affected the representativeness of our sample, but previously published sensitivity analyses of this same data found this effect not to be statistically significant [1].

## Conclusions

Using a longitudinal population-based sample of adolescent and young adult twins, we found no association between adolescent physical activity and the future development of clinically assessed EDs in female twins when adjusted for several factors. However, given our limited sample size, caution is recommended when interpreting the results. Future studies should use longitudinal design with larger population-based samples of all genders to evaluate the association of physical activity and EDs.

## What is already known on this subject?

The relationship between physical activity and the onset of EDs is unclear. Few longitudinal studies have been conducted in the field and these studies have suggested that physical activity is associated with future EDs.

## What does this study add?

We found no association between leisure-time physical activity in adolescence and future development of EDs in female participants.

### Supplementary Information

Below is the link to the electronic supplementary material.

Supplementary file1 (DOCX 39 KB)

## Data Availability

Due to the consent given by study participants and the ease of identifying twins, data cannot be made publicly available. Data are available through the Institute for Molecular Medicine Finland (FIMM) Data Access Committee (DAC) for authorized researchers who have IRB/ethics approval and an institutionally approved study plan. For more details, please contact the FIMM DAC (fimm-dac@helsinki.fi).
