# Supplementary material for: Adolescent leisure-time physical activity and eating disorders: a longitudinal population-based twin study
Source: Eat Weight Disord. 2024 Jun 8;29(1):40. doi: 10.1007/s40519-024-01670-8 (PMC11162369; doi:10.1007/s40519-024-01670-8)
Supplement: Supplementary file 1 — Supplementary file1 (DOCX 39 KB) [file 40519_2024_1670_MOESM1_ESM.docx]

**Adolescent Leisure-Time Physical Activity and Eating Disorders: a Longitudinal Population-Based Twin Study**

**Eating and Weight Disorders-Studies on Anorexia, Bulimia and Obesity**

Nadja Anis, Anna Keski-Rahkonen, Sara Kaartinen, Yasmina Silén, Jaakko Kaprio and Sari Aaltonen

Corresponding author: Nadja Anis, Department of Public Health, P.O. Box 20, FI-00014 University of Helsinki, Helsinki, Finland. Email: nadja.anis@helsinki.fi

| **Supplementary Table 1.**  **Physical activity of the female twins based on the original physical activity categories. The table contains the number of female twins who had the information of both physical activity and future eating disorders (ED) (yes/no).** | |
| --- | --- |
| **Physical activity at age 12** | N=683 (percentage) |
| not at all | 219 (32.1%) |
| two to three times in six months | 42 (6.1%) |
| two to three times a month | 60 (8.8%) |
| two to three times a week | 306 (44.8%) |
| just about every day | 56 (8.2%) |
| **Physical activity at age 14** | N=653 (percentage) |
| not at all | 14 (2.1%) |
| less than once a month | 23 (3.5%) |
| one to two times a month | 63 (9.6%) |
| about once a week | 132 (20.2%) |
| two to three times a week | 207 (31.7%) |
| four to five times a week | 90 (13.8%) |
| just about every day | 124 (19.0%) |
| **Physical activity at age 17** | N=571 (percentage) |
| not at all | 39 (6.8%) |
| less than once a month | 26 (4.6%) |
| one to two times a month | 44 (7.7%) |
| about once a week | 105 (18.4%) |
| two to three times a week | 193 (33.8%) |
| four to five times a week | 78 (13.7%) |
| just about every day | 86 (15.1%) |

**Supplementary Material 1**

**Covariates**

Based on the previous literature on eating disorders [1, 2] and physical activity behavior [3], as well as based on our testing on confounding effects, we considered the following covariates in the present study:

Adolescent academic performance

Twins’ academic performance was assessed and reported by teachers as a grade point average at ages 12 and 14. At age 17, the twins reported their student status, which was used as a measure of academic performance at that stage of their lives. For each study wave, we used the measure of academic performance from the same study wave as a covariate in our analyses.

Parental educational level

Parental education levels were measured by maternal and paternal questionnaires at baseline when the twins were 12 years old. We used the educational level of the more highly educated parent classified into four categories (compulsory education, vocational secondary education, academic secondary education, tertiary education).

Maternal and paternal leisure-time physical activity

Parental physical activity levels were measured by maternal and paternal questionnaires at baseline when the twins were 12 years old. Similarly to the twins, both parents reported their monthly physical activity frequency but also the duration and intensity of physical activity sessions. By using these items, we were able to calculate leisure-time metabolic equivalent of task hours per day (ltMETh/d) values to measure the total energy expenditure of the parents’ physical activity. The ltMETh/d values were calculated as follows: physical activity frequency × mean duration × mean intensity [4]. For example, 4 ltMETh/d corresponds to about 60 minutes of walking per day.

References:

1. Goodman, A., Heshmati, A., & Koupil, I. (2014). Family history of education predicts eating disorders across multiple generations among 2 million Swedish males and females. *PloS One*, *9*(8), e106475. https://doi.org/10.1371/journal.pone.0106475

2. Sundquist, J., Ohlsson, H., Winkleby, M. A., Sundquist, K., & Crump, C. (2016). School Achievement and Risk of Eating Disorders in a Swedish National Cohort. *Journal of the American Academy of Child and Adolescent Psychiatry*, *55*(1), 41–46.e1. https://doi.org/10.1016/j.jaac.2015.09.021

3. Bauman, A. E., Reis, R. S., Sallis, J. F., Wells, J. C., Loos, R. J., Martin, B. W., & Lancet Physical Activity Series Working Group. (2012). Correlates of physical activity: Why are some people physically active and others not? *Lancet (London, England)*, *380*(9838), 258–271. https://doi.org/10.1016/S0140-6736(12)60735-1

4. Aaltonen, S., Kaprio, J., Kujala, U. M., Pulkkinen, L., Rose, R. J., & Silventoinen, K. (2018). The Interplay between Genes and Psychosocial Home Environment on Physical Activity. *Medicine and Science in Sports and Exercise*, *50*(4), 691–699. https://doi.org/10.1249/MSS.0000000000001506

| **Supplementary Table 2. Wald test results on the covariate characteristics of the study participants by future eating disorder (ED) status** | | | |
| --- | --- | --- | --- |
|  |  |  |  |
| **Characteristics** | **Future ED status**  **ED dx** | **No ED dx** |  |
|  | n (%) | n (%) | **p-value** |
| **School performance at age 12** |  |  | 0.37 |
| grade point average under 6 | 0 (0%) | 0 (0%) |  |
| grade point average 6-7  grade point average 7-8 | 0 (0%)  41 (34.8%) | 17 (3.1%)  190 (34.8%) |  |
| grade point average 8-9 | 72 (61.0%) | 311 (57.0%) |  |
| grade point average better than 9 | 5 (4.2%) | 28 (5.1%) |  |
|  |  |  |  |
| **School performance at age 14** |  | | 0.73 |
| grade point average under 6 | 1 (1.4%) | 0 (0%) |  |
| grade point average 6-7 | 3 (4.1%) | 30 (6.9%) |  |
| grade point average 7-8 | 15 (20.6%) | 109 (24.9%) |  |
| grade point average 8-9 | 45 (61.6%) | 235 (53.7%) |  |
| grade point average better than 9 | 9 (12.3%) | 64 (14.6%) |  |
| **Student status at age 17** |  | | 0.32 |
| not studying currently | 1 (2.2%) | 14 (2.7%) |  |
| in vocational school | 9 (19.6%) | 137 (26.0%) |  |
| in upper secondary school | 36 (78.3%) | 376 (71.4%) |  |
| **Parental educational level**^†^ |  | | 0.41 |
| compulsory education | 26 (21.7%) | 156 (27.7%) |  |
| vocational secondary education | 38 (31.7%) | 157 (27.8%) |  |
| academic secondary education | 37 (30.8%) | 164 (29.1%) |  |
| tertiary education | 19 (15.8%) | 87 (15.4%) |  |
|  | Mean (SD) | Mean (SD) |  |
| **Maternal physical activity (ltMETh/d)** | 3.00 (3.30) | 2.40 (2.71) | 0.06 |
| **Paternal physical activity (ltMETh/d)** | 2.80 (3.22) | 2.42 (3.20) | 0.27 |

ED, Eating disorder; dx, diagnosis; ltMETh/d, leisure-time metabolic equivalent of task hours per day;SD, standard deviation;

^†^ educational level of the more highly educated parent

| **Supplementary Table 3. Longitudinal association of female adolescents’ physical activity and restrictive eating disorders^a^ (EDs) based on Cox proportional hazards model (including unadjusted and adjusted model).** | | | | | | | | | | |
| --- | --- | --- | --- | --- | --- | --- | --- | --- | --- | --- |
|  | **Unadjusted model^b^** | | | | | **Adjusted model^c^** | | | | |
|  | **Future restrictive ED** | |  |  |  | **Future restrictive ED** | |  |  |  |
|  | **ED dx** | **No ED dx** | **HR** | **95% CI** | ***P*-value** | **ED dx** | **No ED dx** | **HR** | **95% CI** | ***P*-value** |
| **Physical activity at age 12** | **N=73** | **N=562** |  |  |  | **N=64** | **N=466** |  |  |  |
| Rarely | 34 | 270 | 1 |  |  | 33 | 232 | 1 |  |  |
| Regularly | 33 | 248 | 1.03 | 0.63-1.69 | 0.91 | 26 | 201 | 0.84 | 0.49-1.46 | 0.54 |
| Daily | 6 | 44 | 1.07 | 0.45-2.53 | 0.88 | 5 | 33 | 0.91 | 0.35-2.37 | 0.84 |
| **Physical activity at age 14** | **N=52** | **N=561** |  |  |  | **N=33** | **N=377** |  |  |  |
| Rarely | 13 | 208 | 1 |  |  | 11 | 138 | 1 |  |  |
| Regularly | 20 | 176 | 1.82 | 0.90-3.72 | 0.10 | 10 | 120 | 1.09 | 0.42-2.81 | 0.86 |
| Daily | 19 | 177 | 1.68 | 0.84-3.33 | 0.14 | 12 | 119 | 1.23 | 0.54-2.80 | 0.62 |
| **Physical activity at age 17** | **N=21** | **N=525** |  |  |  | **N=18** | **N=463** |  |  |  |
| Rarely | 5 | 199 | 1 |  |  | 5 | 172 | 1 |  |  |
| Regularly | 11 | 175 | 2.47 | 0.86-7.06 | 0.09 | 9 | 154 | 1.60 | 0.57-4.48 | 0.37 |
| Daily | 5 | 151 | 1.31 | 0.38-4.51 | 0.67 | 4 | 137 | 0.84 | 0.24-2.98 | 0.78 |
| ED, eating disorder; dx, diagnosis; CI, confidence interval; HR, hazard ratio  ^a^=Anorexia nervosa (AN), Atypical AN, Restrictive syndrome, Purging disorder  **^b^**= model without covariates  **^c^=**  model adjusted for covariates (all ages) and at age 14 also stratified by parental education level  Covariates: school performance (grade point average at age 12, grade point average at age 14) or student status (student status at age 17), maternal physical activity (ltMETh/d), paternal physical activity (ltMETh/d), parental education level based on the level of the more highly educated parent | | | | | | | | | | |

| **Supplementary Table** **4. Longitudinal association of female adolescents’ physical activity and non-restrictive eating disorders^a^ (EDs) based on Cox proportional hazards model (including unadjusted and adjusted model).** | | | | | | | | | | |
| --- | --- | --- | --- | --- | --- | --- | --- | --- | --- | --- |
|  | **Unadjusted model^b^** | | | | | **Adjusted model^c^** | | | | |
|  | **Future non-restrictive ED** | |  |  |  | **Future non-restrictive ED** | |  |  |  |
|  | **ED dx** | **No ED dx** | **HR** | **95% CI** | ***P*-value** | **ED dx** | **No ED dx** | **HR** | **95% CI** | ***P*-value** |
| **Physical activity at age 12^*^** | **N=46** | **N=562** |  |  |  | **N=39** | **N=466** |  |  |  |
| Rarely | 15 | 270 | 1 |  |  | 14 | 323 | 1 |  |  |
| Regularly | 25 | 248 | 1.78 | 0.89-3.56 | 0.10 | 20 | 201 | 1.78 | 0.85-3.72 | 0.13 |
| Daily | 6 | 44 | 2.31 | 0.93-5.79 | 0.07 | 5 | 33 | 2.12 | 0.68-6.57 | 0.19 |
| **Physical activity at age 14^*^** | **N=39** | **N=561** |  |  |  | **N=30** | **N=377** |  |  |  |
| Rarely | 11 | 208 | 1 |  |  | 8 | 138 | 1 |  |  |
| Regularly | 10 | 176 | 1.07 | 0.46-2.48 | 0.87 | 9 | 120 | 1.12 | 0.44-2.82 | 0.82 |
| Daily | 18 | 177 | 1.86 | 0.85-4.07 | 0.12 | 13 | 119 | 1.64 | 0.66-4.11 | 0.29 |
| **Physical activity at age 17** | **N=24** | **N=525** |  |  |  | **N=22** | **N=463** |  |  |  |
| Rarely | 10 | 199 | 1 |  |  | 9 | 172 | 1 |  |  |
| Regularly | 7 | 175 | 0.80 | 0.31-2.08 | 0.64 | 6 | 154 | 0.75 | 0.27-2.11 | 0.59 |
| Daily | 7 | 151 | 0.93 | 0.36-2.38 | 0.87 | 7 | 137 | 0.95 | 0.38-2.35 | 0.91 |
| ED, eating disorder; dx, diagnosis; CI, confidence interval; HR, hazard ratio  ^a^=Bulimia nervosa (BN), Binge eating disorder (BED), BN of low frequency and/or limited duration, BED of low frequency and/or limited duration, Subthreshold BN/BED, Other, Insufficient information  **^b^**= model without covariates  **^c^=**  model adjusted for covariates (all ages) and at ages 12 and 14 also stratified by parental education level  Covariates: school performance (grade point average at age 12, grade point average at age 14) or student status (student status at age 17), maternal physical activity (ltMETh/d), paternal physical activity (ltMETh/d), parental education level based on the level of the more highly educated parent | | | | | | | | | | |
